# Effects of BPA, BPS, and BPF on Oxidative Stress and Antioxidant Enzyme Expression in Bovine Oocytes and Spermatozoa

**DOI:** 10.3390/genes13010142

**Published:** 2022-01-14

**Authors:** Mimi Nguyen, Reem Sabry, Ola S. Davis, Laura A. Favetta

**Affiliations:** Reproductive Health and Biotechnology Lab, Department of Biomedical Sciences, Ontario Veterinary College, University of Guelph, Guelph, ON N1G 2W1, Canada; mnguye06@uoguelph.ca (M.N.); rsabry@uoguelph.ca (R.S.); odavis@uoguelph.ca (O.S.D.)

**Keywords:** bisphenol A, bisphenol S, bisphenol F, oocytes, spermatozoa, oxidative stress

## Abstract

Bisphenol A (BPA) and its analogs, bisphenol S (BPS) and bisphenol F (BPF), might impact fertility by altering oxidative stress pathways. Here, we hypothesize that bisphenols-induced oxidative stress is responsible for decreased gamete quality. In both female (cumulus-oocyte-complexes—COCs) and male (spermatozoa), oxidative stress was measured by CM-H_2_DCFDA assay and key ROS scavengers (SOD1, SOD2, GPX1, GPX4, CAT) were quantified at the mRNA and protein levels using qPCR and Western blot (COCs)/immunofluorescence (sperm). Either gamete was treated in five groups: control, vehicle, and 0.05 mg/mL of BPA, BPS, or BPF. Our results show elevated ROS in BPA-treated COCs but decreased production in BPS- and BPF-treated spermatozoa. Additionally, both mRNA and protein expression of SOD2, GPX1, and GPX4 were decreased in BPA-treated COCs (*p* < 0.05). In sperm, motility (*p* < 0.03), but not morphology, was significantly altered by bisphenols. SOD1 mRNA expression was significantly increased, while GPX4 was significantly reduced. These results support BPA’s ability to alter oxidative stress in oocytes and, to a lesser extent, in sperm. However, BPS and BPF likely act through different mechanisms.

## 1. Introduction

A growing body of evidence suggests that environmental contaminants have the potential to negatively impact animal and human health. Endocrine-disrupting chemicals (EDCs) are known to interfere and mimic endogenous endocrine function [1]. Among EDCs, bisphenol A (BPA) has been used for decades in the plastics industry. BPA is found ubiquitously in food packaging, personal care items, cash register receipts, and medical equipment, just to name a few. Its widespread use makes avoiding exposure nearly impossible; to date, BPA has been detected in aquatic environments, sewage, tap water, soil, dust, and air, posing a danger to humans and wildlife alike [2]. BPA’s primary route of exposure is through the diet as it leaches from plastics to food [3]. Leakage is enhanced by heat, contact with acidic or basic substances, and repeated use [3]. Unsurprisingly, BPA has been repeatedly detected in several biological samples, such as placental tissue, serum, follicular fluid, amniotic fluid, and urine [4].

Concerns surrounding BPA use arise from its interactions with hormonal receptors. As a weak xenoestrogen, BPA is able to bind to the classical nuclear estrogen receptors ERα and ERβ [5]. As such, while BPA has been associated with detrimental effects in immune, nervous, and cardiovascular systems, a particular concern is its effects on reproductive function, given its strong reliance on the hypothalamic–pituitary–gonadal axis [5]. Recent studies suggest that BPA can affect steroidogenesis in both females [6,7,8,9] and males [10,11,12].

Given the evident health risks of BPA, many countries have restricted or completely banned its use, leading to an industry shift to “BPA-free” products containing BPA analogs such as bisphenol S (BPS) and bisphenol F (BPF) [13]. However, these analogs remain unregulated, and insufficient data exist to support their safety [13]. BPS and BPF are structurally similar to BPA and, as such, are expected to exhibit comparable physiological effects on reproduction. Although this field of research is still in its early stages, current evidence suggests that the analogs’ toxicity is equal or even greater than BPA [14,15,16,17].

BPA appears to disturb both female and male reproductive function even at extremely low exposure levels. The affinity of BPA to ER can be 100,000-fold weaker than that of estradiol, though its potency increases when estradiol is extremely low [4,5]. Although there are various alternative pathways in which BPA impairs endocrine function, alteration of oxidative stress remains a key contributor in both male and female infertility [5]. Oxidative stress occurs when the production of reactive oxygen species (ROS) exceeds the protective capacity of its endogenous antioxidant defense mechanisms [18]. ROS refer to a group of oxygen free radicals, such as superoxide anions and hydrogen peroxide, that are formed as by-products of the mitochondrial respiratory chain [19]. ROS are highly reactive but can be stabilized via oxidizing biological macromolecules and organelles, resulting in cellular damage [20]. In contrast, antioxidants, including superoxide dismutase 1 (SOD1) and superoxide dismutase 2 (SOD2), glutathione peroxidase 1 (GPX1) and glutathione peroxidase 4 (GPX4), and catalase (CAT) can provide protection against ROS by neutralizing free radicals [20]. While some ROS production is crucial in both female (folliculogenesis, ovulation, embryonic development) [21] and male (capacitation, hyperactivation, acrosomal reaction) [22] reproduction processes, the excessive presence of ROS can result in increased oxidative stress, germ cell apoptosis, and, ultimately, infertility.

Developing oocytes are under strict regulation by steroid hormones in order to gain competency. Oxidative damage during maturation is speculated to be one of the main causes of abnormal meiosis, decreased rate of fertilization, and an overall decline in embryonic viability [19]. Several studies have linked BPA toxicity and risen oxidative stress in non-reproductive tissues, such as the liver [23] and the heart [24]. One of the first studies to suggest that BPA modulates the generation of ROS in the oocyte was conducted by Wang et al. [25], who found that ROS levels significantly increased after 26 h of in vitro culture of porcine oocytes. mRNA expression of several oxidative stress-related genes was analyzed, and a significant increase in SOD1 was found in the BPA treatment group, indicating that BPA-treated porcine oocytes underwent oxidative stress. Consequently, oocytes treated with BPA had reduced polar body extrusion, altered spindle morphology, abnormal chromosome alignment, and increased rates of apoptosis, leading to reduced oocyte maturation [25]. These findings are aligned with data from our laboratory and additional literature, where bovine oocytes displayed increased spindle abnormalities and chromosome misalignment following BPA treatment [26,27,28]. The majority of studies have been conducted on porcine, mice, or rat models, and little information is available on BPS and BPF, two of BPA’s most common analogs, and their potential effects in altering intracellular oxidative stress in oocytes and sperm.

Spermatozoa are even more vulnerable to the effects of oxidative stress. In the final stage of spermatogenesis, the majority of the cytoplasm is shed in preparation of fertilization. As such, mature spermatozoa lack critical repair mechanisms needed to relieve oxidative damage [22]. Additionally, sperm membranes are rich in polyunsaturated fatty acids, making sperm a viable target for oxidative damage via lipid peroxidation. In turn, this triggers a sequence of inflammatory events, resulting in loss of membrane integrity, increased permeability, structural DNA damage, and apoptosis [29]. Once a lipid peroxide radical is formed, it initiates a series of oxidation events that can affect over 50% of the spermatozoa plasma membrane. The by-products of lipid peroxidation include malondialdehyde (MDA) and 4-hydroxynonenal (4-HNE), which further damage sperm DNA [30]. An in vivo study in mice found a significant increase in lipid peroxidation and ROS levels as well as a decrease in GPX activity in the testes following BPA treatment [31]. They also found that BPA-treated mice had decreased sperm concentration and motility [31].

Given constraints of human oocyte and sperm use, the bovine model is a well-suited translational model in reproductive toxicology for humans. Several physiological similarities exist between cattle and humans, such as the process of folliculogenesis, single ovulation, and the size of both female and male gametes [32,33,34]. Additionally, BPA exposure is not limited to humans. Livestock animals are highly and continuously exposed to BPA from their diet because of contaminated soil and water and through farming equipment such as water pipes and milking apparatus [34]. As a lipophilic chemical, BPA can accumulate in adipose tissue, be secreted into milk fat, and persist in dairy products [35]. As such, farm animals are equally, if not more, susceptible to the endocrine-disrupting effects of bisphenols [36].

In this study, not only do we aim to confirm the effects of BPA in altering oxidative stress levels in gametes, but we also investigate whether BPA’s analogs, BPS and BPF, affect oocytes and sperm by increasing oxidative stress. We hypothesize that in vitro exposure to BPA, BPS, and BPF increases oxidative stress levels due to alteration of antioxidant enzyme expression, which ultimately affects the reproductive capability of both female and male gametes.

## 2. Materials and Methods

### 2.1. Reagents

All chemicals and media were purchased from Sigma Aldrich (Oakville, ON, Canada) unless otherwise specified.

### 2.2. Cumulus-Oocyte-Complex (COC) Collection and Maturation

Bovine (*Bos taurus*) ovaries were obtained from local abattoirs (Cargill Meat Solutions, Guelph, ON, Canada, and Highland Packers, Stoney Creek, ON, Canada). COCs were collected by aspirating follicles into a medium of 1 M HEPES buffered F-10 Ham supplemented with 2% steer serum (Gibco; Whitby, ON, Canada), heparin (2 IU/mL), and penicillin/streptomycin (10,000 IU/mL/10,000 IU/mL) (Invitrogen; Burlington, ON, Canada). After aspiration, oocytes were matured in vitro using the protocol previously established in our lab [37,38]. To summarize, pools of 40 COCs were matured in 80 μL micro-drops of in vitro HEPES-buffered TCM199 maturation medium (S-IVM; M4530) supplemented with 2% steer serum, sodium pyruvate, follicle-stimulating hormone (FSH) (Vetoquino; Cambridge, ON, Canada), estradiol (5 μg/mL; E2785), and luteinizing hormone (LH) (NIH; San Diego, CA, USA). Treatment groups included control (2.5 mL S-IVM + H), vehicle (2.5 mL S-IVM + H with 2.5 μL 0.1% ethanol), and the three bisphenol treatments: BPA (239658), BPS (43034), and BPF (51453) at a concentration of 0.05 mg/mL (2.5 mL S-IVM + H with 2.5 μL of the respective bisphenol diluted in 0.1% ethanol). Estradiol was used as a physiological control as bisphenols compete with estradiol for ERs. Micro-drops were covered with mineral oil and matured in a humidified incubator (38.5 °C, 5% CO_2_). After 24 h of maturation, the COCs were either snap-frozen in liquid nitrogen for qPCR and Western blot use or fertilized to produce in vitro blastocysts. Maturation was assessed in 10 oocytes for each treatment group (*n* = 10) by identifying the extrusion of the first polar body under fluorescent microscopy while quantifying the total ROS amount.

### 2.3. Dose–Response Curve and In Vitro Embryo Production

Dose–response curves for BPA and BPS have been conducted previously in our lab by Sabry and colleagues [37], but a curve for BPF has yet to be established in our experimental model. Therefore, to determine the appropriate dose for BPF-treated bovine oocytes, a dose-dependent curve was performed to assess cleavage and blastocyst rates. The doses used for the curve include the 0.05 mg/mL (equivalent to the lowest observed adverse effect level (LOAEL) dose of BPA in the bovine model) 10× and 100× lower and 10× higher doses. Thus, pools of 40 COCs were matured for 24 h, as previously described, in the five treatment groups, washed, transferred to 80 μL micro-drops of IVF-TALP + BSA and covered with mineral oil. COCs were fertilized using frozen-thawed *Bos taurus* semen (Semex; Guelph, ON, Canada) from the same bull of established in vitro fertilization capability. The highest quality spermatozoa were isolated using a swim-up method 1 h prior to fertilization, as described by Saleh et al. [38]. Each micro-droplet, containing 20 treated COCs, received a concentration of 1 × 10^6^ sperm cells/mL and was incubated (38.5 °C, 5% CO_2_) for 18 h.

Presumptive zygotes (PZs) were mechanically stripped using a micropipette, washed, and cultured in 30 μL droplets of synthetic oviductal fluid (SOF) medium with 15% BSA, 2% FBS, 88.6 µg/mL sodium pyruvate, 2% non-essential amino acids, 1% essential amino acids, and 0.5% gentamicin (IVC medium). Micro-drops were covered with mineral oil and incubated in a low oxygen (5% O_2_) incubator. Cleavage rate and blastocyst rates were determined at 48 h and 8 days post-fertilization, respectively. Cleavage rate was determined by comparing the number of embryos cleaved to the total number of oocytes fertilized, while blastocyst rate was determined by comparing the number of blastocysts formed to the number of embryos cleaved.

### 2.4. Spermatozoa Preparation and Evaluation of Motility and Morphology

Cryopreserved semen of one bull with known fertility and proven in vitro fertilization capability (Semex; Guelph, ON, Canada) was thawed in a 37 °C water bath for 30 s. The bull used in this study is representative of the results obtained on three other bulls (*n* = 4, including the bull here shown). We previously established that the bull sample used here showed consistent results under the same experimental conditions as the results obtained on frozen and fresh semen of three other individual bulls of known and proven fertilization capability. In fact, when motility (both by Makler Counter Chamber and the Sperm Class Analyzer-SCA^®^ CASA System software) and morphology (by microscopy) were analyzed on all four bulls at the same time, no differences were observed among the four bulls investigated in technical triplicates [39].

To isolate motile sperm from extender debris and dead spermatozoa, semen samples were washed using a discontinuous Percoll density gradient of 500 μL 45% over 500 μL 90% of Percoll solution (GE17-0891-01). Percoll solution was made by combining 250 µL of 90% Percoll solution with 250 µL HEPES/Sperm TALP and phenol red to distinguish between the layers. Semen was layered on top of the Percoll gradient and centrifuged at 600× *g* for 20 min. Supernatant was removed and discarded, leaving a sperm pellet that was resuspended in 1 mL HEPES/Sperm-TALP medium containing 15% BSA, NaCl, KCl, Na_2_HPO_4_·12H_2_O, CaCl·2H_2_O, and MgCl·6H_2_O. Sperm cells were pelleted again by centrifugation at 600× *g* for an additional 7 min, followed by the removal of the supernatant. Sperm pellets were resuspended in 50 μL of HEPES/Sperm-TALP and equally divided amongst 5 treatment groups: control (1 mL HEPES/Sperm-TALP), vehicle (1 mL HEPES/Sperm-TALP + 1 μL 0.1% ethanol), BPA (1 mL HEPES/Sperm-TALP + 1 μL 50 mg/mL BPA stock), BPS (1 mL HEPES/Sperm-TALP + 1 μL 50 mg/mL BPS stock), and BPF (1 mL HEPES/Sperm-TALP + 1 μL 50 mg/mL BPF stock). Thus, spermatozoa were treated at a physiologically relevant dose of 0.05 mg/mL (BPA LOAEL dose) of their respective bisphenol, mimicking treatments in COC experiments. Samples were incubated in a humidified environment for 4 h. Incubation time was determined based on a pilot time-dependent experiment to investigate the effects of BPA on motility, and it is also supported by the data of Li et al. [40] from human spermatozoa. Treatment groups were then centrifuged at 600× *g* for 7 min to pellet the sperm. Supernatant was removed, and sperm pellets were resuspended and collected for (i) motility and/or morphology assessments or (ii) ROS quantification or (iii) RNA extraction and PCR analysis.

Examination of motility and morphology was conducted based on the World Health Organization (WHO) laboratory manual for the examination and processing of human semen [40]. Both morphology and motility were assessed by one analyst and under blinded conditions to avoid treatment bias. For morphology, 10 μL of washed, well-mixed spermatozoa was smeared onto a pre-warmed microscope slide using a feathering technique, as described in the WHO laboratory manual [41]. Once air-dried, slides were fixed in 3:1 methanol/acetic acid for one hour, washed once in Milli-Q water, and stained using a Giemsa solution (5 mL Giemsa stock to 45 mL Milli-Q water) for 15 min. Morphology was manually determined by assessing at least 100 spermatozoa per treatment group under high magnification. Defects were categorized based on head, midpiece, and tail abnormalities. Head defects include pyriform heads, tapered heads, or detached heads, while midpiece anomalies include bent necks and proximal or distal cytoplasmic droplets. Lastly, tail defects are bent, coiled, or shortened tails.

Motility was assessed by pipetting 10 μL of washed spermatozoa to a pre-warmed Makler counting chamber to be immediately observed under a microscope at 200× magnification. At least 100 spermatozoa per treatment group per replicate were assessed for motility and were categorized into progressive, non-progressive, and immotile. Progressive motility refers to spermatozoa moving actively, either linearly or in a large circle, while non-progressive motility occurs when spermatozoa are swimming in small circles or moving in place without forward movement. In contrast, immotility refers to the lack of tail movements overall [41].

### 2.5. Measurement of Reactive Oxygen Species (ROS)

Intracellular ROS production was measured using the fluorescent probe 5-(and-6)-chloromethyl-20,70-dichlorodihydrofluorescein diacetate, acetyl ester (CM-H_2_DCFDA—Invitrogen, C6827). CM-H_2_DCFDA is a general oxidative stress indicator that fluoresces green in correlation to the amount of ROS detected; 50 µg of CM-H_2_DCFDA was prepared by dissolving 86.5 µL of 100% ethanol to a concentration of 1 mM. All ROS detection experiments were done in low-light conditions. Both female and male gametes were stained using CM-H_2_DCFDA to determine oxidative stress after bisphenol treatment.

For the measurement of ROS in COCs, the same groups described earlier (control, vehicle, and the three bisphenols at the BPA LOAEL dose) were used, along with a positive control (10 µL of 100% hydrogen peroxide—H_2_O_2_) and a negative control (counterstained only). After 24 h of maturation, COCs were transferred into 500 µL hyaluronidase (2 mg/mL) to remove cumulus cells, along with gentle aggravation using a micropipette. Denuded oocytes were washed 3× in sterile phosphate-buffered saline (PBS) (Multicell, Wisent Bioproducts; Quebec, Canada) with 0.01% polyvinyl alcohol (PVA) and incubated in pre-warmed PBS/PVA with freshly prepared CM-H_2_DCFDA at a concentration of 5 µM for 30 min at atmospheric conditions. Oocytes were counterstained using Hoechst (blue), incubated for an additional 15 min, washed 3× in PBS/PVA, and mounted on a slide with DAKO Fuorescence mounting medium (Aligent Technologies; Mississauga, ON, Canada). Oocytes were immediately observed under an Olympus FV1200 confocal microscope at 40× objective using laser wavelengths of 405 nm for Hoechst (blue) and 488 nm for Alexa-Fluor 488 (green). Fluorescence intensity of 10 COCs per treatment group was determined using ImageJ software. The green fluorescence channel was isolated, and each COC was selected one at a time to measure its cell area and integrated density. Background intensity was also measured and accounted for in this technique. Fluorescence intensity was measured by calculating the corrected total cell fluorescence (CTCF), as previously described [42], using the following formula:

CTCF = integrated density − (area of selected cell × mean fluorescence of background readings).

For the male gamete, spermatozoa were prepared, as described earlier, in treatment groups of control, vehicle, BPA, BPS, and BPF (at the BPA LOAEL dose of 0.05 mg/mL). Additional groups for ROS quantification were the positive and negative controls as in the oocyte experiments. After 4 h in their respective treatment groups, sperm were pelleted via centrifugation at 600× *g* for 7 min and resuspended in 100 µL of pre-warmed HEPES/Sperm-TALP with 1 µM of freshly prepared CM-H_2_DCFDA. Samples were incubated for 15 min in the dark at 37 °C. After incubation, 1 µL of propidium iodide (PI, stock 1 mg/mL) was added to each group and incubated for an additional 15 min. Samples were then washed, transferred to slides, and fixed as previously described. A coverslip was applied to slides with DAKO fluorescent mounting medium, sealed, and stored at 4 °C. Slides were imaged under an inverted fluorescent microscope (Leica DM IRE2) at 60× objective. At least 100 sperm per group per replicate were quantified using the corrected total cell fluorescence (CTCF) technique after determining fluorescence intensity using ImageJ software, as described above.

### 2.6. RNA Extraction and Reverse Transcription

For COCs, RNA extraction was conducted as described by Saleh et al. [38]. In brief, pools of 35 COCs per treatment group underwent RNA extraction using the Qiagen RNeasy Plus Micro Kit (Qiagen; Toronto, ON, Canada) according to the manufacturer’s guidelines. Samples were snap-frozen at −80 °C until reverse transcription.

For sperm, total RNA was extracted using the Macherey-Nagel Nucleospin^®^ miRNA Kit (Valencienner, Düren, Germany). All centrifugations were done at 11,000× *g* for 1 min unless specified. Sperm pellets containing approximately 100 × 10^6^ sperm cells were resuspended in 300 µL lysis buffer (Buffer ML), mixed, homogenized by sonication, and incubated at room temperature (RT) for 15 min; 300 µL of the lysate was transferred to a NucleoSpin^®^ filter column and centrifuged; 100% ethanol was added to each tube, vortexed and incubated at RT for 5 min. Flow-through was loaded onto a NucleoSpin^®^ RNA column and centrifuged. Resulting flow-through was discarded, and the RNA column was treated with 350 µL Membrane Desalting Buffer (MDB) and centrifuged. After DNAse treatment and additional washes, the supernatant was transferred to a NucleoSpin^®^ Protein Removal Column, centrifuged, and 800 µL of binding buffer (Buffer MX) was added. Washes to remove DNA fragments, salts, or other contaminants were performed following the manufacturer’s instructions. Samples were eluted in 100 µL of RNAse-free water for maximal efficiency, concentrated in the Jouan centrifugal evaporator (RCT 60) and vacuum concentrator (RC 1010) (Thermo Scientific; Mississauga, ON, Canada) to a final volume of 20 µL. Samples were snap-frozen at −80 °C until reverse transcription. RNA quantity and quality were assessed using a NanoDrop 2000c (Thermo Scientific; Mississauga, ON, Canada); 250 ng of sperm and COC RNA were reverse-transcribed into cDNA using QuantaBio qScript cDNA SuperMix (VWR; Mississauga, ON, Canada) in the T100 thermal cycler (BioRad; Mississauga, ON, Canada), as described by Sabry et al. [37]. Once transcribed, cDNA samples were stored at −20 °C until used for qPCR.

### 2.7. Quantitative Polymerase Chain Reaction (qPCR)

qPCR was used to determine the mRNA expression of the five antioxidant enzymes (SOD1, SOD2, CAT, GPX1, and GPX4) using a CFX96 Touch Real-Time PCR Detection System (BioRad). cDNA was amplified using the SsoFast EvaGreen Supermix (Biorad, 1725201), as described previously [38]. The primer sequences used for qPCR analysis are specified in Table 1. Efficiencies for each primer set were between 90–110%, as determined by standard curve. Relative changes in mRNA expression were calculated by the efficiency-corrected method (ΔΔCt) using tyrosine 3- monooxygenase/tryptophan 5-monooxygenase activation protein zeta (YWHAZ) and H2A histone family member Z (H2AFZ) as reference genes. YWHAZ and H2AFZ were determined to be unaffected by treatments for both oocytes and sperm based on GeNorm analysis. To account for inter-run variability, a group of 100 COCs was used as a calibrator for the COC experiments, while a pool of five cryopreserved semen straws (1 mL containing 50 million sperm) was used as a calibrator for the sperm experiments, respectively. At least three biological replicates in technical triplicates were quantified. 

### 2.8. Western Blotting

Protein expression of 5 antioxidant enzymes was quantified from pools of 35 COCs by Western blotting, as described by Saleh et al. [38]. To summarize, samples were lysed in RIPA, sonicated, and centrifuged at 12,000× *g* at 4 °C to isolate proteins; 30 µg of protein were loaded to each well and were separated on 12% polyacrylamide gels using an XCell SureLock Mini-Cell Electrophoresis System (Invitrogen; Burlington, ON, Canada) for 125 V for 2 h. Proteins were transferred onto a nitrocellulose membrane (Bio-Rad, 1620115) at 45 V for 2 h on ice. Membranes were stained with Ponceau S to ensure adequate protein transfer, then blocked in 5% skim milk in TBST for 1 h. Afterwards, membranes were incubated overnight at 4 °C in a primary antibody: SOD2 at 1:1000 (Invitrogen, PA1-31072), CAT at 1:800 (Invitrogen, PA5-23246), and GPX1 at 1:800 (Invitrogen, 711797). These three antioxidants were chosen as significant changes were observed at the mRNA level.

Protein levels were detected using a 1:5000 dilution of the anti-rabbit IgG HRP-linked (Cell Signalling Technology; Whitby, ON, Canada; 70735) secondary antibody for 1 h at room temperature. Membranes were imaged on a ChemiDoc XRS + Imaging System (Bio-Rad) after a 5 min incubation in Clarity Western ECL Blotting Substrate (Bio-Rad 170–5060). β-actin antibody (Cell Signalling Technology, 4967) at a 1:200 dilution overnight at 4 °C was used as the loading control on all blots. Densitometric analysis was performed using Bio-Rad Image Lab software, and protein levels are expressed as a ratio to β-actin.

### 2.9. Immunofluorescent Staining of Sperm

Immunofluorescence was used to evaluate the protein quantity and localization of three antioxidant enzymes (SOD1, GPX1, and GPX4) in sperm. Washed spermatozoa were incubated 4 h in the same treatment groups as described previously, with a positive (H_2_O_2_) and negative control (Hoechst counterstain). Sperm were then fixed in 4% paraformaldehyde (PFA) for 30 min at room temperature and stored in 2% PFA in PBS at 4 °C until used; 5 µL from each sample was applied to multi-welled microscope slides, air-dried, and then a few drops of the methanol/acetic acid fixative were applied to the slide. Once air-dried, the slides were washed, and fixed sperm were permeabilized by 0.5% Triton ×100 + 0.1% sodium citrate in 1× PBS. Sperm was blocked using 1× PBS supplemented with 5% normal donkey serum (NDS) for 1 h at RT. Slides were incubated in the primary antibody, either SOD1 at 1:5000 (Invitrogen, Burlington, ON, Canada; PA5-23245), GPX1 at 1:800 (Invitrogen, 711797), or GPX4 at 1:1000 (Invitrogen, PA5-18545) overnight at 4 °C in a sealed, humidified chamber in the dark. A specific secondary antibody was used at 1:200 dilution (Donkey anti-Rabbit IgG (H + L) Highly Cross-Adsorbed Secondary Antibody, Alexa-Fluor 488 (Invitrogen, ThermoFisher Scientific; Mississauga, ON, Canada; A21206) for SOD1 and GPX1 and Donkey anti-Goat IgG (H + L) Cross-Adsorbed Secondary Antibody, Alexa Fluor 488 (Invitrogen, ThermoFisher Scientific; Mississauga, ON, Canada; A-11055) for GPX4). Slides were incubated in the dark in a humidified chamber for one hour at 37 °C. Then, 10 µL of Hoechst nuclear stain was added for an additional 15 min in the humidified chamber at 37 °C. Once air-dried, slides were covered with coverslips with DAKO fluorescent mounting medium, sealed and stored at 4 °C until imaged using an Olympus FV1200 Confocal Microscope at a 20× and/or 40× objective. Laser wavelengths include 488 nm for Alexa-Fluor 488 (green) and 405 nm for Hoechst (blue).

Fluorescent images were analyzed using ImageJ software. First, Hoechst-stained nuclei (blue) were separated and counted to determine the total number of sperm. Then, localization was determined based on the FITC-staining (green) of sperm. Points of interest for localization included the acrosome, cytoplasm, equatorial band, post-acrosomal sheath, midpiece, and flagella. Intensity of antioxidant expression was determined by averaging the calculated corrected total cell fluorescence of 100 spermatozoa per treatment group in triplicate.

### 2.10. Statistical Analysis

All data sets were analyzed for statistical significance using GraphPad Prism 9 software. Prior to analysis, the normality of data was determined using Kolmogorov–Smirnov and Shapiro–Wilk tests. Normally distributed data sets were analyzed using one-way analysis of variance (ANOVA), while non-symmetric data were analyzed using the Kruskal–Wallis test. Significant data sets were then subjected to further analysis. Parametric data was followed by Tukey’s post hoc test, while non-parametric analysis was followed by Dunn’s multiple comparison test. At least three biological replicates were used in each experiment, and statistical difference was determined at a two-tailed *p*-value < 0.05. Data shown represent the mean ± standard error of the mean (SEM).

## 3. Results

### 3.1. Dose–Response Curve for BPF

**Figure 1:**
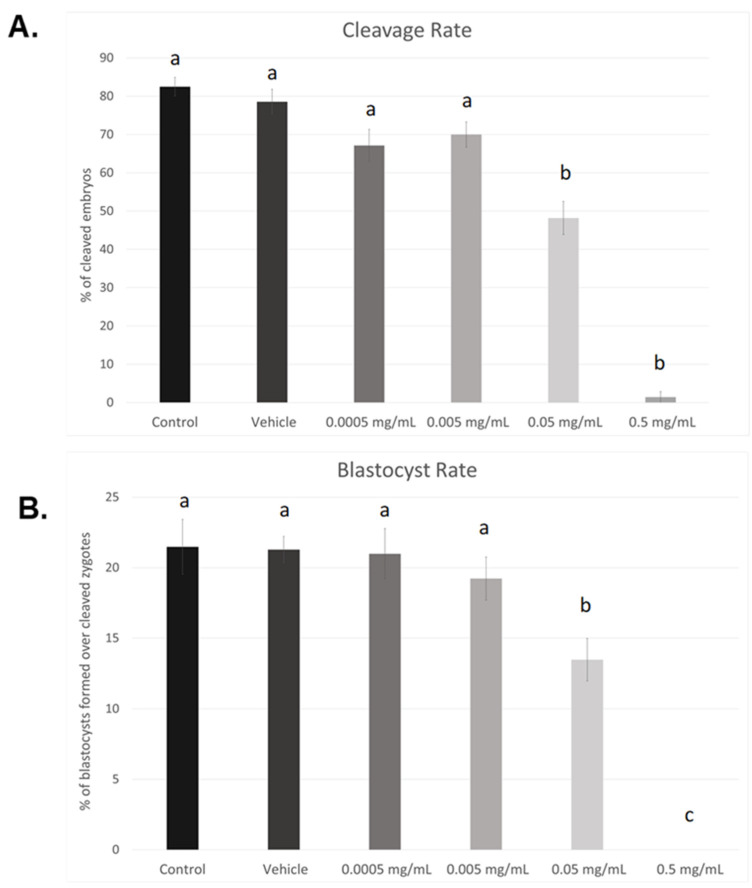
Dose–response curves after in vitro exposure of bovine oocytes to BPF. (**A**) depicts cleavage rates determined 24 h after fertilization. (**B**) represents blastocyst rates measured 8 days post-fertilization. Treatment groups include control (IVM + H media only), vehicle (IVM + H + 0.1% ethanol), and 4 serial dilutions of BPF (0.5 mg/mL, 0.05 mg/mL, 0.005 mg/mL, and 0.0005 mg/mL diluted in 0.1% ethanol and IVM + H media). Different letters indicate significant differences, with b and c representing *p*-values of <0.05 and <0.0001 versus a, respectively. Error bars represent ±SEM.

As seen in Figure 1A, cleavage rates were significant reduced in the 0.05 mg/mL dose (*p* < 0.05) as well as the highest dose of BPF, 0.5 mg/mL (*p* < 0.004). Compared to control and vehicle, no significant changes were observed at lower doses (0.005 mg/mL and 0.0005 mg/mL). This data set was analyzed through the non-parametric Kruskal–Wallis test, followed by Dunn’s multiple comparison test. Of the zygotes that cleaved, a significantly lower percentage progressed to the blastocyst stage when treated with the LOAEL dose (*p* < 0.03). No blastocysts were produced in any of the biological replicates when treated with the highest dose of BPF (*p* < 0.0001). These data were analyzed through the parametric ANOVA test, followed by Tukey’s post hoc test. Overall, these results indicate a dose-dependent effect of BPF on embryonic development. As such, the dose equivalent to the BPA LOAEL dose was chosen for future BPF experiments, as the highest dose used in this dose–response curve is lethal to blastocyst formation. Additionally, this dose is consistent with the doses used for other bisphenols, BPA and BPS, in our lab [37,38].

**Figure 2:**
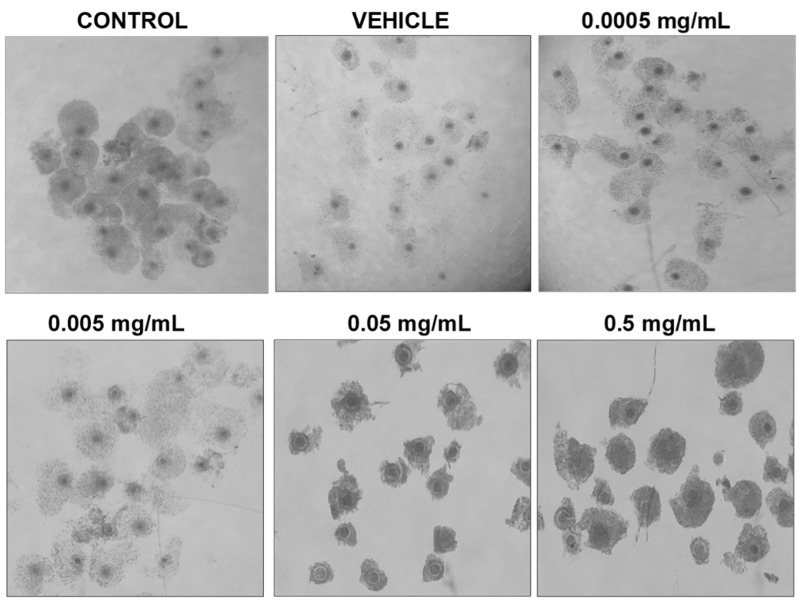
Morphology of cumulus-oocyte-complexes after 24 h of maturation in serial dilutions of BPF. Treatments from left to right include control (IVM + H media only), vehicle (IVM + H + 0.1% ethanol), and 4 concentrations of BPF (0.5 mg/mL, 0.05 mg/mL, 0.005 mg/mL, and 0.0005 mg/mL diluted in 0.1% ethanol and IVM + H media).

Morphologically, differences can be observed after 24 h of maturation in their respective doses of BPF (Figure 2). COCs in control, vehicle, and the lowest doses of BPF have a similar appearance with multilayered cumulus cell expansion and are overall light/transparent in color. However, COCs treated at the LOAEL dose and higher show less cumulus expansion and are darker in appearance. The morphological assessment followed the grading of de Loos et al. [46]. No changes in % of matured oocytes were detected among treatment groups or between treatments and controls and vehicle (data not shown).

### 3.2. Sperm Motility and Morphology

**Figure 3:**
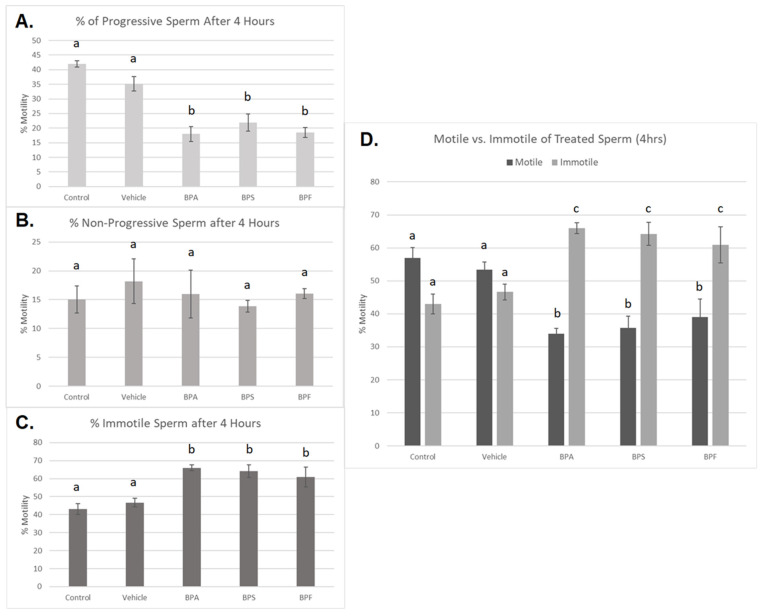
Motility of bovine spermatozoa treated with BPA, BPS, and BPF (0.05 mg/mL) for 4 h. (**A**) represents the % of progressive sperm, (**B**) represents % of non-progressive sperm, and (**C**) represents the % of immotile sperm. (**D**) demonstrates motile (both progressive and non-progressive movement) compared to immotile sperm. Different letters indicate significant differences, with b and c denoting a *p*-value of <0.03. Error bars represent ±SEM.

Treated spermatozoa were categorized into progressive, non-progressive, and immotile sperm. After 4 h of incubation, a significant decrease in progressive motility was observed in all three bisphenol groups compared to controls (*p* < 0.03) (Figure 3A). Additionally, the percentage of immotile sperm was significantly increased in all three bisphenol-treated groups after 4 h (*p* < 0.03) (Figure 3C). Interestingly, no significant changes were seen in the number of non-progressive sperm across the five treatment groups (Figure 3B). This data set was analyzed through the parametric ANOVA test, followed by Tukey’s post hoc test. Overall, when motile sperm, including both progressive and non-progressive movement, was compared to immotile sperm (Figure 3D), all three bisphenols exhibited a significant increase in the amount of immotile sperm after 4 h of incubation.

**Figure 4:**
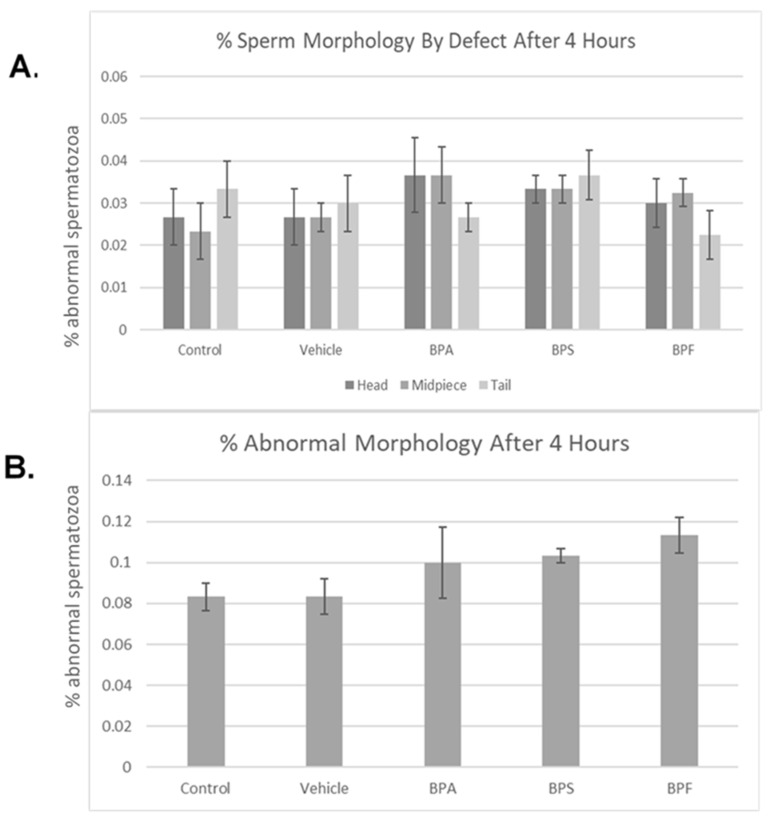
Morphology of bovine spermatozoa after 4 h incubation in BPA, BPS, and BPF treatment. (**A**) depicts the percentage of abnormalities based on defect type (head, midpiece, tail). (**B**) shows the overall percentage of abnormalities found. No significant differences were observed when separated by defect or overall. Error bars represent ±SEM.

Bisphenol treatment did not affect morphology (head, midpiece, tail) (Figure 4A). When defects were combined, no notable changes were seen in the amount of abnormal sperm (Figure 4B). Overall, 4 h of incubation in bisphenol treatment had no effect on morphology, with each group yielding ~10% abnormal sperm. This was analyzed through the parametric ANOVA test, followed by Tukey’s post hoc test. As previously mentioned in Section 2.4 of this manuscript, the bull used in this study is representative of the results obtained on three other bulls (*n* = 4, including the bull here shown).

### 3.3. Oxidative Stress

**Figure 5:**
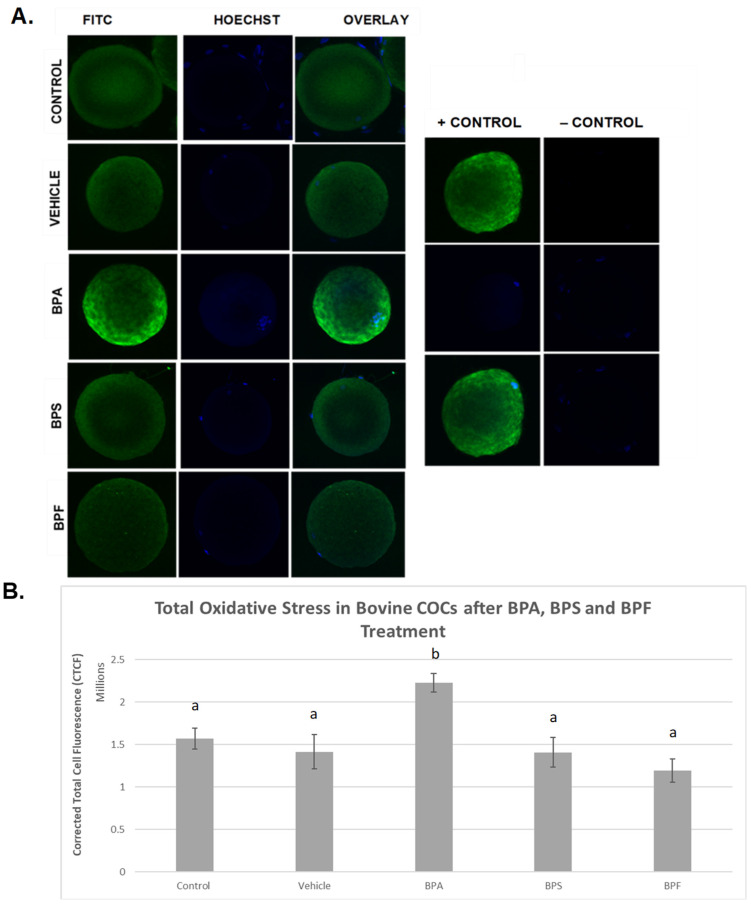
Quantification of ROS as a measure of total oxidative stress after 24 h of maturation in BPA, BPS, and BPF of denuded bovine oocytes. (**A**) denotes oocytes stained with CM-H_2_DCFDA and captured using an Olympus FV1200 confocal microscope and analyzed using ImageJ software. Fluorescence intensity correlates with ROS generation. (**B**) represents the corrected total cell fluorescence (CTCF) of 10 denuded oocytes per group. Different letters indicate significant differences, with b indicating a *p* < 0.05, and error bars are ±SEM.

ROS levels were examined after bisphenol treatment of bovine oocytes as an indicator of total oxidative stress (Figure 5). In the images depicted (Figure 5A), it can be observed that the intensity of green fluorescence in the BPA group is noticeably higher than in all the other groups, indicating increased ROS production in this group. BPS- and BPF-treated oocytes exhibit the same fluorescence as the control and vehicle groups, indicating that the amount of ROS produced is similar. When fluorescence intensity was measured (Figure 5B), trends from observation were confirmed in that the generation of ROS was substantially increased in BPA-treated oocytes compared to controls and all other groups (*p* < 0.05). No significant differences in ROS generation were detected in cells treated with BPS or BPF compared to controls. These data were analyzed through the non-parametric Kruskal–Wallis test, followed by Dunn’s multiple comparison test.

**Figure 6:**
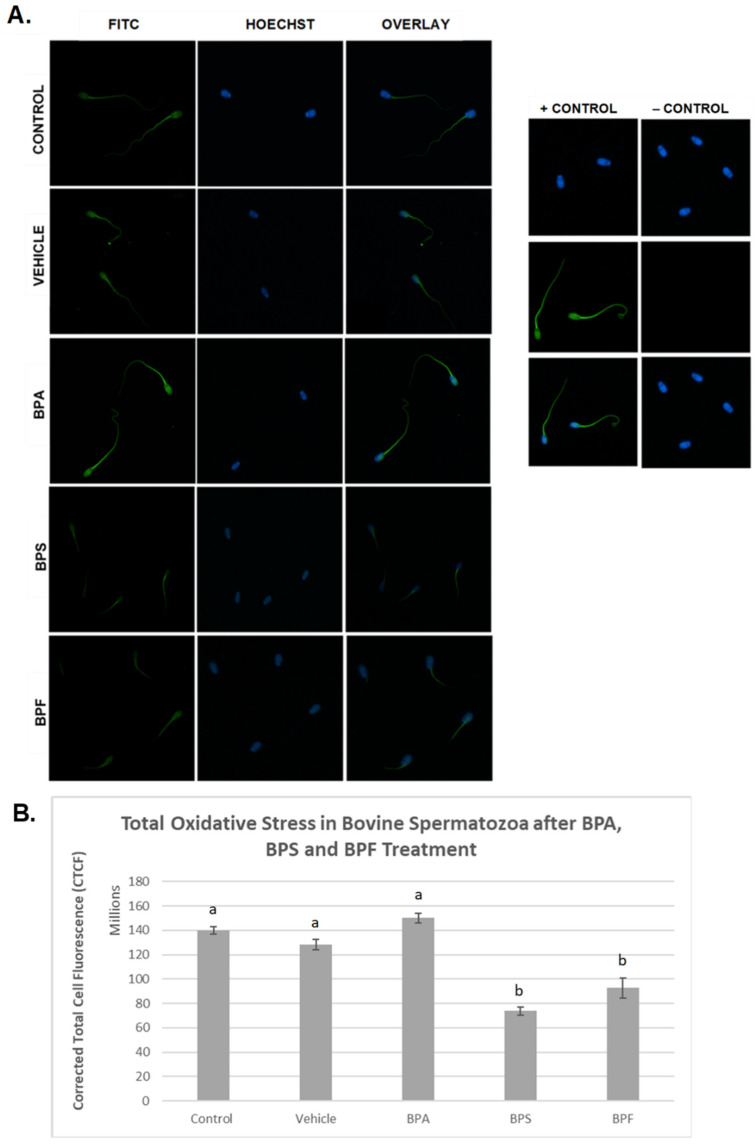
Quantification of ROS after 4 h of incubation in BPA, BPS, and BPF of bovine spermatozoa. (**A**) Sperm was stained with CM-H_2_DCFDA, which fluoresces relative to the amount of ROS produced and is an indicator of total oxidative stress. (**B**) Corrected total cell fluorescence (CTCF) is shown. b indicates a *p* < 0.03 versus a, and error bars represent ±SEM.

In spermatozoa (Figure 6A), the images indicate the same trend in the BPA-treated group, where fluorescent intensity in the BPA-treated groups was noticeably higher compared to all other groups. However, when intensities were quantified, no significant differences were observed between BPA treatment and controls. Unexpectedly, the other bisphenol-treated groups, BPS and BPF, had significantly decreased levels of ROS (*p* < 0.03) compared to other groups. This data set was analyzed through the non-parametric Kruskal–Wallis test, followed by Dunn’s multiple comparison test.

### 3.4. mRNA Expression in Treated Oocytes and Sperm

The mRNA of five antioxidant enzymes was quantified in oocytes and sperm treated with BPA, BPS, and BPF relative to housekeeping genes YWHAZ and H2AFZ.

**Figure 7:**
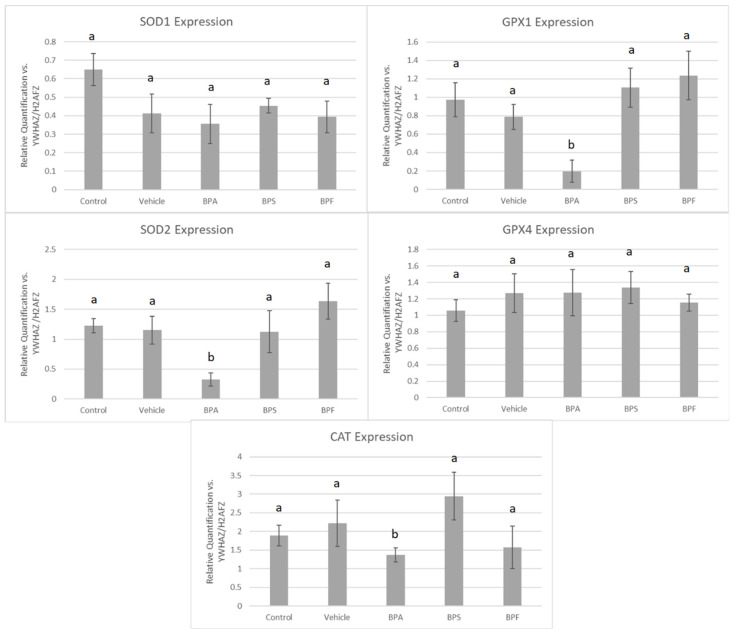
mRNA expression of five antioxidant enzymes in bovine COCs after 24 h of maturation in BPA, BPS, and BPF treatments (0.05 mg/mL). Quantification is relative to reference genes YWHAZ and H2AFZ. Different letters indicate significant differences, with b indicating a *p*-value <0.05 versus a, and error bars represent ±SEM.

In COCs, mRNA expression of SOD2, GPX1, and CAT was significantly reduced (*p* < 0.05) in the BPA treatment group. These changes were not observed for SOD1 and GPX4 in BPA-treated COCs. The mRNA expression of all five antioxidants was unaffected by BPS and BPF treatment (Figure 7). This was analyzed through the parametric ANOVA test, followed by Tukey’s post hoc test.

**Figure 8:**
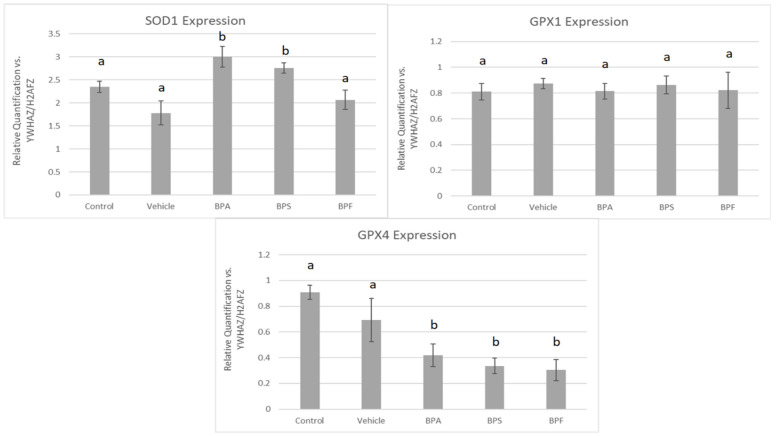
mRNA expression of three antioxidants (SOD1, GPX1, GPX4) in bovine spermatozoa after 4 h of incubation of BPA, BPS, and BPF at a dose of 0.05 mg/mL. Different letters indicate significant differences, with b denot a statistical significance of *p* < 0.05 versus a. Error bars represent ±SEM.

In sperm, a significant increase in mRNA expression for SOD1 was observed in BPA and BPS groups but not in the BPF group (*p* < 0.05). In contrast, GPX4 expression was significantly decreased in all three bisphenol groups compared to control and vehicle (*p* < 0.05). Lastly, GPX1 expression was unaffected by bisphenol treatment as there were no significant differences across the five groups (Figure 8). These data were analyzed through the parametric ANOVA test, followed by Tukey’s post hoc test. SOD2 and CAT mRNA could not be quantified in any treatment group, indicating that either SOD2 and CAT are not expressed in sperm or they are expressed below the detection limits of the technique used.

### 3.5. Protein Expression of COCs after BPA, BPS, and BPF Treatment

**Figure 9:**
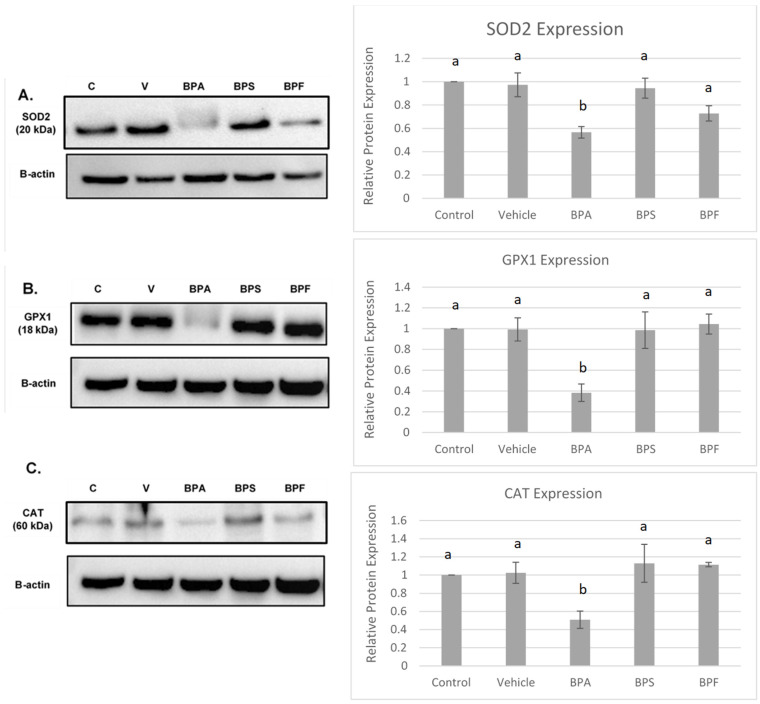
Western blot of SOD2 (**A**), GPX1 (**B**), CAT (**C**) protein expression in bovine COCs. For each antioxidant enzyme, a representative blot is seen on the left and densitometric analysis relative to the loading control, β-actin, is seen on the right. Western blot data represents 4 biological replicates. Different letters indicate significant differences: b denotes statistical significant difference versus a (*p* < 0.05). Error bars represent ±SEM.

Protein levels of the antioxidant enzymes SOD2, CAT, and GPX4 were quantified in bovine COCs relative to the loading control, β-actin. The effects of BPA, BPS, and BPF on antioxidant expression can be observed in Figure 9.

All three antioxidants (SOD2, GPX1, and CAT) were statistically reduced by BPA treatment (*p* < 0.05) compared to control and vehicle. This data set was analyzed through the parametric ANOVA test, followed by Tukey’s post hoc test.

### 3.6. Protein Expression and Localization of Sperm

**Figure 10:**
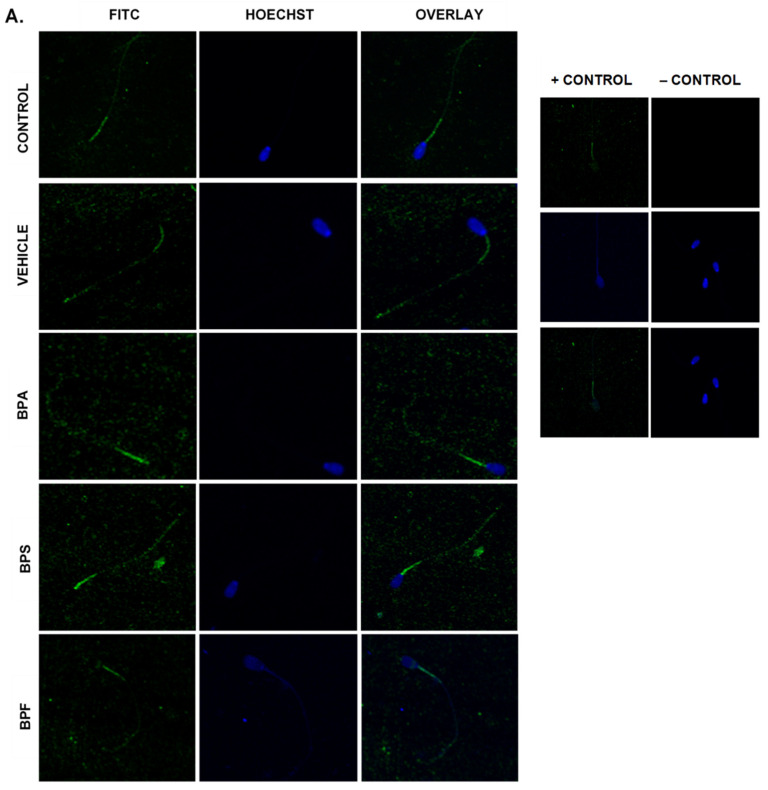

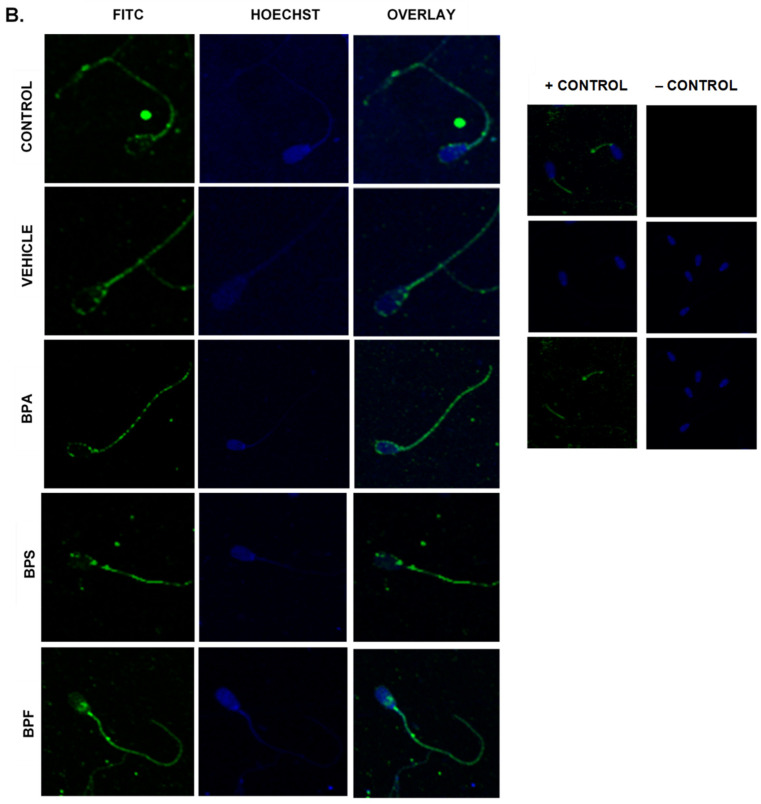

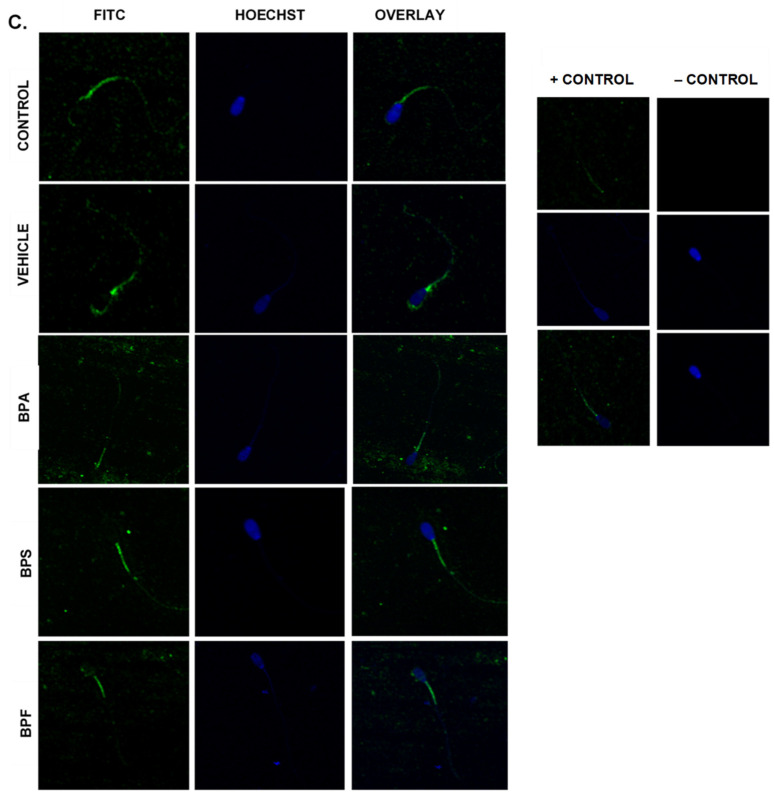

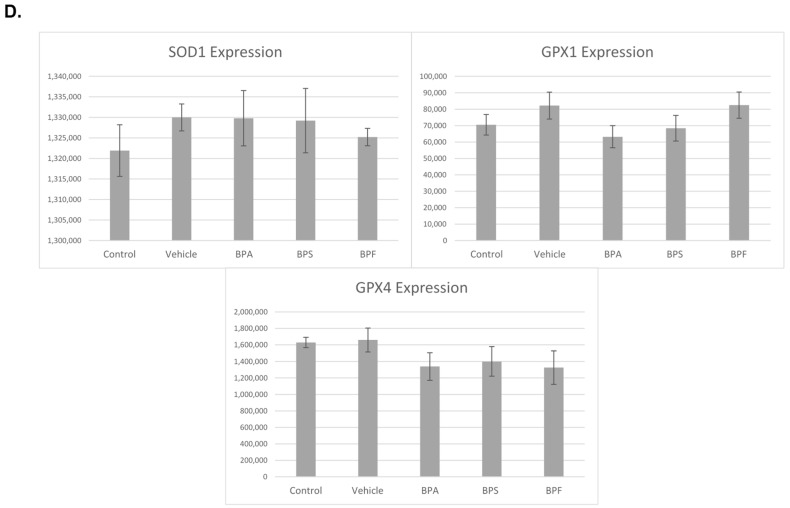
Relative protein expression of SOD1 (**A**), GPX1 (**B**), and GPX4 (**C**), determined via immunofluorescent staining in bovine spermatozoa treated with BPA, BPS, and BPF. Images were taken at 40× objective using the Olympus FV1200 confocal microscope at laser wavelengths of 405 nm for Hoechst (blue) and 488 nm for Alexa-Fluor 488 (FITC) (green). Localization of respective antioxidants can be determined based on the location of the Alexa-Fluor 488 present. (**D**) demonstrates the corrected total cell fluorescence (CTCF) of SOD1, GPX1, and GPX4 in bovine spermatozoa determined using ImageJ software. Fluorescent intensity of Alexa-Fluor 488 correlates with protein expression. No significant differences were detected.

Immunofluorescent staining was conducted on treated bovine spermatozoa to determine protein expression and localization of antioxidant enzymes SOD1, GPX1, and GPX4, as seen in Figure 10A–C. The present study found that bisphenol treatment did not alter protein expression of SOD1, GPX1, or GPX4. Analysis was conducted through the parametric ANOVA test, followed by Tukey’s post hoc test. For the SOD1 enzyme, protein was observed in the midpiece of the sperm. The expression of protein in control and vehicle groups compared to the three bisphenols remained unchanged in the midpiece, indicating that bisphenol treatment did not alter the localization of the SOD1 enzyme. For GPX1 and GPX4, expression was also observed in the midpiece as well as around the head of the sperm (acrosomal area). Like SOD1, the localization of GPX1 remained consistent in all treatments and was unaffected by bisphenol exposure. However, compared to control and vehicle, GPX4 was absent from the acrosomal area and was present solely in the midpiece of treated sperm. Therefore, our results indicate that BPA, BPS, and BPF affect the location of expression of GPX4 enzyme in the head of the sperm but not in the midpiece.

## 4. Discussion

Bisphenols are well-established as endocrine disruptors. However, the full range of mechanisms through which they produce their effects has yet to be fully elucidated. One of the suggested mechanisms by which bisphenols impair oocyte maturation and sperm fertilization potential is through the alteration of oxidative stress pathways. The work presented here aimed to determine the effects of BPA, BPS, and BPF on oocytes’ and sperm’s oxidative stress levels. Oxidative stress occurs due to the imbalance between protective antioxidants and damaging reactive oxygen species; while oxidative stress has been well-documented to negatively affect both oocytes and sperm, bisphenol-induced oxidative stress in gametes and early development has yet to be fully characterized.

Prior to investigating bisphenol-induced changes in oxidative stress, a BPF dose–response curve was established. Presently, no governmental regulations exist regarding safe dosage for BPF for humans or animals alike. Therefore, the LOAEL dose for BPA of 0.05 mg/mL was adopted as BPF is used in similar concentrations to BPA in the manufacturing industry [9]. As bisphenol exposure occurred solely during in vitro maturation, effects on cleavage and blastocyst rates demonstrate the effects of BPF on oocyte competence. Oocyte competence refers to the ability of the oocyte to successfully mature in a manner that allows for fertilization and development into a viable embryo [47]. The process of oocyte maturation is tightly regulated, and disturbances during this time could affect its developmental potential. Our results indicate significant declines in both cleavage and blastocyst rates at the 0.05 mg/mL and 0.5 mg/mL doses, indicating that these doses affect the maturation process [48]. To further support the idea that BPF affects oocyte maturation in parallel to its counterparts BPA and BPS, we can compare our findings to dose–response curves for BPA and BPS. Saleh et al. [38] exposed bovine COCs to the same concentrations described in this study. At the highest dose, exposure to BPA, BPS, and BPF resulted in no blastocyst formation. However, while no cleavage was also documented in the highest dose of BPA, a very small percentage (<10%) of cleaved embryos was observed after treatment with BPS or BPF at the same dose. Additionally, the 0.05 mg/mL dose resulted in decreased cleavage and blastocyst rates after all three bisphenols’ exposure; however, significance was only observed for BPA and BPF. These findings suggest that the effects of BPF may be more detrimental to oocyte development than BPS but less than BPA (BPA > BPF > BPS). This is supported in the literature on other cell types, as shown by Molina-Molina et al. [49], who suggested that BPS has weaker estrogenic effects on human breast cancer MCF-7 cells than BPA and BPF, which have comparable potency. Additionally, BPS appears to be less likely to induce mitochondrial-related apoptosis than BPA and BPF in human erythrocytes [50].

In spermatozoa, we observed a significant decrease in progressive motility after treatment with all three bisphenols. Although the experiments here presented have been conducted in technical replicates on an individual bull, we have established that this bull is representative of at least three other bull semen samples previously analyzed (*n* = 4) [39]. Motility is vital to the reproductive potential of the male gamete to fertilize the oocyte as sperm must swim to penetrate both the cumulus layer and the zona pellucida [51]. Elevated ROS levels in the male reproductive tract have been repeatedly documented in individuals with poor motility (asthenozoospermia) and in cases of male idiopathic infertility. Although sperm cells actively generate ROS in order to induce changes associated with sperm capacitation, an overproduction of ROS can lead to excess lipid peroxidation. Since sperm membranes have notably high levels of polyunsaturated fatty acids, they are particularly vulnerable to free radical attack, leading to the direct inhibition of sperm movement [52]. ROS generation and oxidative stress can also lead to a loss in mitochondrial membrane potential, which is considered a potential regulator of sperm motility [53]. Thus, we speculate that a decrease in sperm motility likely arises from ROS accumulation and subsequent oxidative stress. ROS accumulation in sperm can also lead to DNA damage, increasing the fragmentation levels in the nucleus [52]. Sperm DNA damage has been linked to lower fertilization capability and higher miscarriage and developmental abnormalities in offspring [54].

No significant differences in non-progressive motility were observed. Non-progressive motility refers to the movement of sperm without forward progression, such as swimming in small circles, flagellar beat, or movement of the head by flagellar force [41]. In particular, circular movement can be a sign of hyperactivated motility and capacitation, which is a series of biochemical transformations that occurs in sperm in preparation of fertilization within the female reproductive tract [55]. Capacitation-like changes can occur in cryopreserved semen due to the frequent stressors that occur during the freezing process [56]. However, since the specific type of non-progressive motility was not determined in this study, it is still unclear whether bisphenols affect capacitation status in cryopreserved bovine sperm. However, a study by Li et al. [57] suggests that BPA exposure for 4 h decreased capacitation and the acrosomal reaction in human sperm. Sperm from this study also exhibited decreased protein tyrosine phosphorylation but did not show any differences in intracellular calcium concentration after bisphenol exposure. BPA’s effects in sperm could be mediated by ROS generation, which has been speculated to play a role in tyrosine phosphorylation by activating the cyclic adenosine monophosphate (cAMP) pathway [58]. In particular, low levels of ROS are involved in the stimulation of adenylyl cyclase activity, activation of protein kinase A, and the inhibition of tyrosine phosphatase activity [52]. Thus, perhaps BPA elicits an overgeneration of ROS, resulting in oxidative stress that affects capacitation in sperm.

Our findings suggest that BPA-treated oocytes had a significant increase in ROS production compared to all other groups when treated at the LOAEL dose, indicating that BPA exposure results in increased oxidative stress in bovine oocytes. These findings are in alignment with the literature in other species, such as a study by Park et al. [26] that found increased ROS generation after in vitro BPA treatment of porcine oocytes. Similar increases in ROS generation were also observed in mouse oocytes after BPA exposure [57]. In sperm, we found an unexpected significant decrease in oxidative stress after BPS and BPF exposure. We expected BPA, BPS, and BPF treatment to result in elevated ROS generation and subsequent oxidative stress; however, BPS and BPF might affect oxidative stress to a lesser extent than BPA. In a study by Castellini et al. [59], human spermatozoa treated with BPS and BPF in vitro had no significant changes in mitochondrial ROS compared to control samples. Differences between Castellini et al. [59]’s results and the present study could potentially be explained by differences in detection techniques and experimental conditions. The decrease in ROS generation after BPS or BPF exposure in sperm suggests that these analogs may not initiate pro-oxidative or pro-apoptotic mechanisms in the same manner as BPA in the male gamete. Sperm is highly susceptible to oxidative stress, particularly in the mitochondria. When cells are stressed, the mitochondria release cytochrome C to initiate apoptosis; this process has been well-documented to be upregulated by BPA in porcine embryos [27], rat spermatocytes [12], and mice testes [60]. However, in mature sperm, the mitochondria are found in the midpiece, outside of the cytoplasm, where cytochrome C cannot readily enter the sperm heads. Thus, it has been hypothesized that apoptosis in sperm, leading to a loss of sperm motility and oxidative DNA damage, is initiated through ROS production [61].

Antioxidants play an important role in mediating ROS generation. Not all ROS are detrimental; in fact, ROS have functional roles in both oocyte and sperm, as well as during embryonic development [62]. Changes in expression of SOD, GPX, and CAT could negatively affect the gamete’s ability to counteract ROS, leading to elevated oxidative stress. Our results demonstrate a clear imbalance between fewer antioxidants and more ROS during bisphenol exposure. Interestingly, decreased mRNA expression was found at three different stages of the oxidative stress pathway. SOD2 is a key mitochondrial enzyme that catalyzes the conversion of free radicals into oxygen and hydrogen peroxide, which is then reduced to water by CAT and GPX1. CAT is absent from mammalian mitochondria and therefore catalyzes hydrogen peroxide in the cytosol, while GPX1 is found most abundantly in the mitochondria [63]. A significant increase in SOD1 mRNA expression following BPA and BPS exposure, as well as a prominent decrease in GPX4 mRNA expression for all three bisphenols, was detected in sperm. These results are expected as mature spermatozoa extrude the majority of their cytoplasm during spermiogenesis, rendering the male gamete almost transcriptionally and translationally silent [64]. Thus, spermatozoa have low levels of antioxidant enzymes and fewer DNA repair mechanisms in the cytosol to begin with [65]. Additionally, the observed decreases in mRNA expression in sperm align with findings in the literature. For example, in vivo exposure to BPA in mice resulted in decreased activity of CAT [26] and GPX [31,66]. SOD1 is found in the cytosol, while GPX4 is found both mitochondrially and in the nucleus [63]. SOD is typically regarded as a protective enzyme in male reproductive cells, playing a role in maintaining sperm viability. Its activity has been reported to be positively correlated with a sperm cell’s ability to withstand the stress of cryopreservation [67]. Interestingly, GPX4 specifically targets phospholipid hydroperoxides, which are produced abundantly in the sperm membrane [63,68].

In COCs, the protein levels of SOD2, GPX1, and CAT were consistent with mRNA results, strengthening the effects of BPA, but not BPS and BPF, in altering oxidative stress. In sperm, bisphenol treatment did not affect the protein expression of SOD1 and GPX1. These findings matched mRNA results for GPX1, where no statistically significant differences were observed between groups. Statistically significant increases in SOD1 mRNA expression following BPA and BPS exposure are not reflected at the protein level. Lastly, immunofluorescence of GPX4 in sperm shows that GPX4 was localized to the midpiece and the acrosome of bovine spermatozoa; this was expected, given that GPX4 plays a structural role in the mitochondrial capsule [69]. Interestingly, compared to control and vehicle, there appeared to be a loss of GPX4 expression around the acrosome in all three bisphenol groups. The acrosome, found on the outer membrane of the sperm head, is highly sensitive to ROS as it contains an electron-dense region needed for the acrosomal reaction and zona pellucida penetration [70]. The overall decline in GPX4 expression, along with the lack of protein expression in the acrosomal area after BPA, BPS, and BPF exposure, may play a role in the decline of progressive motility after bisphenol treatment.

Reproductive potential of the mammalian oocyte decreases drastically with advanced maternal age, and a major hypothesis to explain this phenomenon is the free radical theory of aging. According to this theory, the accumulation of ROS within the ovarian environment is a contributor to cellular senescence and deteriorating oocyte quality [47]. This is supported by our previous study in granulosa cells, showing that all three bisphenols at low and BPA LOAEL doses significantly increased the production of reactive oxygen species as well as significant increases in antioxidant expression as an initial acute response to bisphenol exposure [71]. The primary site of ROS production in the oocyte is the mitochondria. Functional mitochondria are imperative for normal oocyte function as these organelles are responsible for ATP production, regulation of calcium homeostasis, and cellular metabolism in both oocytes and early embryos [57]. Mitochondrial dysfunction might play a role in how BPA induces oxidative stress in the female gamete. Thus, bisphenol-induced oxidative stress could potentially facilitate a premature aging phenotype, indicative of infertility [72].

We speculate that discrepancies between mRNA and protein expression after BPA exposure arise due to protein degradation. Proteins involved in oxidative phosphorylation, including antioxidants, have been shown to be the first to be affected by ROS and degrade at a faster rate [73]. The mitochondria are considered the major source of intracellular ROS in all three experimental types as they produce ATP for the cells through oxidative phosphorylation. During this process, high-energy electrons progress through the electron transport chain to be accepted by oxygen, which is reduced by water through a complex called cytochrome c oxidase (complex IV) [74]. However, some molecules escape capture by complex IV and are subsequently released as anion superoxide, a highly unstable ROS, which then elicits oxidative protein damage. Since repair of these proteins is limited to cysteine and methionine oxidation, which themselves are susceptible to oxidative stress, damaged proteins are typically eliminated via the Lon protease in the mitochondrial matrix or the proteasome in the cytosol [74]. Further support for the rapid degradation of these proteins comes from the fact that some oxidative damage is irreversible, leading to impaired function or the complete inactivation of the protein that is to be eliminated [74]. Thus, mitochondrial dysfunction resulting from BPA exposure could be responsible for the differences in mRNA and protein expression of antioxidants found in our experiments. Additionally, oxidative stress has been shown to cause both physical and chemical defects in RNA, such as strand breaks and nucleoside base removal. As mentioned previously, 8-oxoG formation occurs regularly as a consequence of oxidative stress; a study by Barciszewski et al. [75] found over 20 oxidized bases in RNA after inducing ROS, with the majority being 8-oxoG. These modifications to the mRNA sequence initiate the generation of short polypeptides due to premature translational termination [76] as well as ribosomal inactivation during protein synthesis [77,78]. Based on our results, it could be speculated that mRNA expression of antioxidants was also upregulated to overcompensate for the early termination of protein synthesis. Future studies in this field could investigate post-transcriptional changes in gametes after bisphenol exposure.

Finally, while antioxidant proteins appeared unaltered by bisphenol exposure in sperm, the activity of the antioxidants is still unknown. Proteins are considered a key target of oxidants due to the high content of the number of oxidative-sensitive amino acid side chains [79]. Under stress conditions, proteins can undergo a series of post-translational modifications; while some reactions occur intentionally and reversibly to regulate redox protein activity, proteins can also undergo several irreversible side-chain modifications such as carbonylation, thiol overoxidation, and di-tyrosine modifications [79]. These irreversible reactions lead to the fragmentation, oligomerization, and degradation of the protein, often inducing a secondary stressor on protein regulation [80]. Modified proteins are particularly vulnerable to mutations and tend to be inactivated or eliminated as they are no longer able to perform their functions. For example, mutations in SOD1 caused by oxidative stress result in enzyme inactivation and misfolding [81]. Other enzymes, such as glyceraldehyde dehydrogenase (GAPDH) in glycolysis, have been shown to undergo enzyme inactivation as a consequence of oxidative stress, leading to the rapid depletion of ATP in *E. coli* [80]. Therefore, enzyme inactivation could explain why we see increased oxidative stress in sperm but a lack of change in the protein expression of the antioxidants. Thus, future studies should investigate the enzymatic activity of antioxidants after bisphenol exposure.

## 5. Conclusions

In conclusion, this study contributes to our understanding of alternative mechanisms of action through which BPA, BPS, and BPF affect oocyte maturation and spermatozoa fertilization potential. Although BPA is known to elicit its effects by binding to the estrogen receptor, our study suggests that oxidative stress may play a role in its deleterious effects on both female and male gamete quality. Based on our results, BPS and BPF do not induce oxidative stress at the same potency as BPA and likely act through different mechanisms.

## Figures and Tables

**Table 1:** Primer sequences.

Gene Symbol	Gene Name	Accession #	Primer Sequence (5′–3′)	Source
SOD1	Superoxide dismutase 1	NM_174615.2	F: AAGATGAAGAGAGGCATGTTGGA R: GATGGCAACACCGTTTTTGTC	[43]
SOD2	Superoxide dismutase 2	NM_201527.2	F: TCTGTTGGTGTCCAAGGCTC R: AGCAGGGGGATAAGACCTGT	
CAT	Catalase	NM_1035386.2	F: CTATGGCCTCCGCGATCTTT R: CGTGAGGCCAAACCTTGGTA	
GPX1	Glutathione peroxidase 1	NM_174076.3	F: CGGGTTCGAGCCCAACT R: GCGCCTTCTCGCCATTC	
GPX4	Glutathione peroxidase 4	NM_1753024.3	F: TGTGGTTTACGGATCCTGGC R: CCCTTGGGCTGGACTTTCAT	[44]
YWHAZ	tyrosine 3-monooxygenase/tryptophan 5-monooxygenase activation protein zeta	NM_174814.2	F: GCATCCCACAGACTATTTCC R: GCAAAGACAATGACAGACCA	[37]
H2AFZ	H2A histone family, member Z	NM_174809.2	F: CTCACCGCAGAGGTACTTGAATT R: AGTCCAATTCTTCATCTCCACGA	[45]

## Data Availability

Not applicable.
